# HBK-14 and HBK-15 Do Not Influence Blood Pressure, Lipid Profile, Glucose Level, or Liver Enzymes Activity after Chronic Treatment in Rats

**DOI:** 10.1371/journal.pone.0165495

**Published:** 2016-10-27

**Authors:** Karolina Pytka, Monika Głuch-Lutwin, Joanna Knutelska, Magdalena Jakubczyk, Anna Waszkielewicz, Magdalena Kotańska

**Affiliations:** 1 Department of Pharmacodynamics, Faculty of Pharmacy, Jagiellonian University Medical College, Krakow, Poland; 2 Department of Pharmacobiology, Faculty of Pharmacy, Jagiellonian University Medical College, Krakow, Poland; 3 Department of Bioorganic Chemistry, Chair of Organic Chemistry, Faculty of Pharmacy, Jagiellonian University Medical College, Krakow, Poland; Max Delbruck Centrum fur Molekulare Medizin Berlin Buch, GERMANY

## Abstract

Older and even new antidepressants cause adverse effects, such as orthostatic hypotension, hyper- or hypoglycemia, liver injury or lipid disorders. In our previous experiments we showed significant antidepressant- and anxiolytic-like activities of dual 5-HT_1A_ and 5-HT_7_ antagonists with α_1_-adrenolitic properties i.e. 1-[(2,6-dimethylphenoxy)ethoxyethyl]-4-(2-methoxyphenyl)piperazine hydrochloride (HBK-14) and 1-[(2-chloro-6-methylphenoxy)ethoxyethyl]-4-(2-methoxyphenyl)piperazine hydrochloride (HBK-15). Here, we evaluated the influence of chronic administration of HBK-14 and HBK-15 on blood pressure (non-invasive blood pressure measurement system for rodents), lipid profile (total cholesterol, low density lipoproteins—LDL, high density lipoproteins—HDL, triglycerides), glucose level, and liver enzymes activity (aspartate aminotransferase, alanine aminotransferase, γ-glutamyl transferase). We determined potential antihistaminic (isolated guinea pig ileum) and antioxidant properties (ferric reducing ability of plasma–FRAP, non-protein thiols–NPSH, stable free radical diphenylpicrylhydrazyl—DPPH) cytotoxicity. Our experiments revealed that HBK-14 and HBK-15 did not influence blood pressure, lipid profile, glucose level or liver enzymes activity in rats after 2-week treatment. We also showed that none of the compounds possessed antioxidant or cytotoxic properties at antidepressant- and anxiolytic-like doses. HBK-14 and HBK-15 very weakly blocked H_1_ receptors in guinea pig ileum. Positive results of our preliminary experiments on the safety of HBK-14 and HBK-15 encourage further studies concerning their effectiveness in the treatment of depression and/or anxiety disorders.

## Introduction

Introduced in the late 80s selective serotonin reuptake inhibitors were superior to tricyclic antidepressants, which caused numerous adverse effects. Although the new group of antidepressants had better overall safety and tolerability, it soon became apparent that they might increase LDL levels (e.g. paroxetine [1]), cause orthostatic hypotension (e.g. fluoxetine [2]), hypoglycemia (e.g. fluoxetine [3]) or liver injury (sertraline [4–6]). Therefore, scientists still search for effective drugs without side effects.

Our previous experiments revealed that dual 5-HT_1A_ and 5-HT_7_ antagonists with α_1_-adrenolytic properties i.e. 1-[(2,6-dimethylphenoxy)ethoxyethyl]-4-(2-methoxyphenyl)piperazine hydrochloride (HBK-14) and 1-[(2-chloro-6-methylphenoxy)ethoxyethyl]-4-(2-methoxyphenyl)piperazine hydrochloride (HBK-15) showed significant antidepressant- and anxiolytic-like effects in mice and rats [7,8]. Both compounds lowered blood pressure after acute treatment [8]. However, HBK-14 unlike HBK-15, showed hypotensive properties at antidepressant-like doses.

Here, we aimed to determine if chronic administration of HBK-14 and HBK-15 influenced blood pressure, lipid and carbohydrate profiles, and liver enzymes activity. We also evaluated the potential antioxidant properties of studied compounds, their cytotoxicity and antihistaminic properties.

## Materials and Methods

### Animals

The experiments were performed on male normotensive Wistar rats (Krf: (WI) WU; weight: approx. 200g) and male guinea pigs (Outbred CV, 300-400g). Rats were purchased from Animal Facility at the Faculty of Pharmacy, Jagiellonian University Medical College, Krakow, Poland and guinea pigs from Laboratory Animals Husbandry Maria Staniszewska, Słaboszów, Poland. Animals were kept in plastic cages (3 rats per cage and 2 guinea pigs per cage) at constant room temperature of 22 ± 2°C, with 12:12 h light/dark cycle. During the experiments rodents had free access to standard pellet diet and water (unless stated otherwise—see section Experimental protocol). Each group consisted of 5–6 animals. After the experiments animals were anaesthetized (75 mg/kg thiopental—rats, 37 mg/kg sodium pentobarbital—guinea pigs) and killed by cervical dislocation. All experimental procedures were approved by the Local Ethics Committee for Experiments on Animals of the Jagiellonian University in Krakow, Poland (approval number 103/2015) and cared for in accordance with the Guide to the Care and Use of Experimental Animals [9].

### Drugs

**Fig 1:**
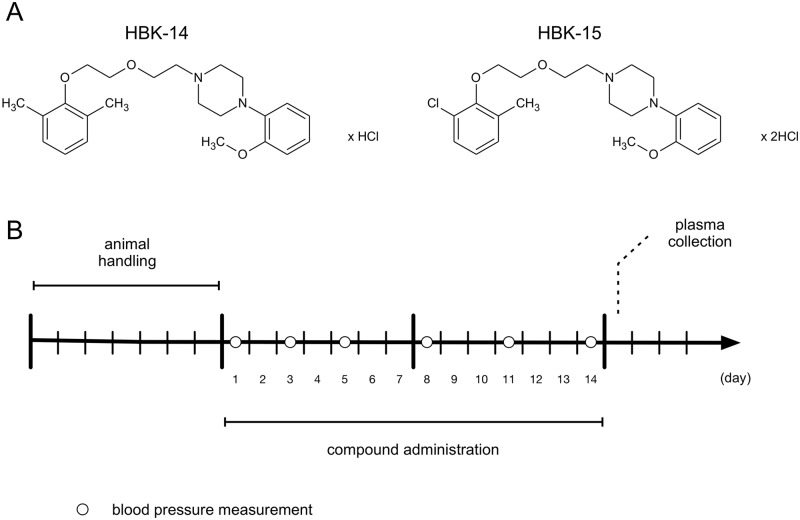
Chemical structures of HBK-14 and HBK-15 (panel A) and experimental protocol—a schematic diagram (panel B). Panel A: HBK-14: 1-[(2,6-dimethylphenoxy)ethoxyethyl]-4-(2-methoxyphenyl)piperazine hydrochloride; HBK-15: 1-[(2-chloro-6-methylphenoxy)ethoxyethyl]-4-(2-methoxyphenyl)piperazine hydrochloride. Panel B: HBK-14 (2.5 and 5 mg/kg), HBK-15 (1.25 and 5 mg/kg), terazosin (5 mg/kg) or saline were administered intraperitoneally to rats for 14 consecutive days. Control groups received 0.9% NaCl (saline).

The studied compounds (Fig 1): 1-[(2,6-dimethylphenoxy)ethoxyethyl]-4-(2-methoxyphenyl)piperazine hydrochloride (HBK-14) and 1-[(2-chloro-6-methylphenoxy)ethoxyethyl]-4-(2-methoxyphenyl)piperazine hydrochloride (HBK-15) were synthesized in the Department of Bioorganic Chemistry, Chair of Organic Chemistry, Faculty of Pharmacy, Jagiellonian University [10]. HBK-14, HBK-15, terazosin and thiopental (Rotexmedica, Germany) were dissolved in saline and administered intraperitoneally (i.p.). Heparin (Polfa S.A., Warsaw) was used as anticoagulant. The control groups received 0.9% NaCl solution. All injections were given in a volume of 1 ml/kg.

### Experimental Protocol

After habituation period, rats were injected with HBK-14 (2.5 or 5 mg/kg), HBK-15 (1.25 or 5 mg/kg), terazosin (5 mg/kg, reference drug) or saline for 14 consecutive days (Fig 1). The doses of studied compounds were based on our previous experiments [7]. The blood pressure was measured three times a week (before and on the 3^rd^, 5^th^, 8^th^, 11^th^ and 14^th^ day of administration). Following the last blood pressure measurement, rodents were deprived of food. 24 hours later rats were anaesthetized, heparinized and plasma was collected.

### Blood pressure measurements

Blood pressure was determined using a non-invasive blood pressure (NIBP) measurement system for rodents (LE 5007 Panlab Harvard Apparatus). First, rats were habituated to handling by an experimenter and to an animal holder for 8–9 days. Blood pressure measurement was performed according to NIBP procedure based on the technique used for arterial blood pressure measurement in humans. The measurement was carried out in rats placed in animal holders and was based on pulse rate measurement on the tail artery every 2 or 3 days. Five readings of systolic and diastolic blood pressure were taken and the mean value was calculated.

### Plasma Collection

After the last administration of the test compounds, animals were deprived of food. On the next day, 20 min after i.p. administration of heparin (1000 j/rat) and thiopental (75 mg/kg), plasma was collected from the left carotid artery of the animals [11].

### Lipid profile and glucose level in plasma

To determine the lipid profile and glucose level in rats’ plasma, standard enzymatic, spectrophotometric tests (Biomaxima S.A. Lublin, Poland) were used. The substrate was decomposed with appropriate enzymes for the relevant product, which was converted to a colored compound. Coloration was proportional to their concentration. The absorbance was measured at a wavelength of 500 (glucose, triglycerides, total cholesterol) or 600 nm (high density lipoproteins—HDL, low density lipoproteins—LDL).

### Liver enzymes activity

To determine the activities of liver enzymes in rat’s plasma, standard spectrophotometric tests (Biomaxima S.A. Lublin, Poland) were used.

#### Aspartate aminotransferase activity

Aspartate aminotransferase activity was determined in plasma samples mixed with L-aspartate, lactate dehydrogenase, malate dehydrogenase, sodium azide, 2-oxoglutarate and reduced nicotinamide adenine dinucleotide (NADH) on the basis of the average change in absorbance per minute. Aspartate aminotransferase catalyzed the transfer of amino group from aspartate to 2-oxoglutarate to form oxaloacetate and glutamate. The oxaloacetate was then reduced by NADH, which resulted in the malate and NAD formation. The rate of change in the absorbance was measured at 340 nm.

#### Alanine aminotransferase activity

Alanine aminotransferase was determined in plasma samples mixed with L-alanine dehydrogenase, lactate, sodium azide, 2-oxoglutarate and NADH on the basis of the average change in absorbance per minute. Alanine aminotransferase catalyzed the transfer of an amino group from alanine to 2-oxoglutarate to form pyruvate and glutamate. The pyruvate was then reduced by NADH, which resulted in the lactate and NAD formation. The rate of change in absorbance was measured at 340 nm.

#### γ-glutamyl transferase activity

The activity of γ-glutamyl transferase was determined in plasma samples mixed with glycyl-glycine and L-γ-glutamyl-3-carboxy-4-nitroanilide on the basis of the average change in absorbance per minute. γ-glutamyl transferase catalyzed the transfer of γ-glutamyl group of L-γ-glutamyl-3-carboxy-4-nitroanilide to the glycyl glycine, with subsequent formation of L-γ-glutamyl-glycyl glycine and yellow colored 5-amino-4-nitrobenzoate. The rate of change in the absorbance was measured at a wavelength of 405 nm.

### Ferric reducing ability of plasma (FRAP)

A modified method of Benzie and colleagues [12] was adopted for the FRAP. The stock solutions included 300 mM acetate buffer (3.1 g of C_2_H_3_NaO_2_·3H_2_O and 16 ml of C_2_H_4_O_2_), pH 3.6, 10 mM 2,4,6- tripyridyl-s-triazine (TPTZ) solution in 40 mM HCl, and 20 mM FeCl_3_·6H_2_O solution. The working solution was prepared by mixing 10 parts of acetate buffer, 1 part of TPTZ solution, and 1 part of FeCl_3_·6H_2_O solution. A portion of 300 μl of the FRAP solution was mixed with 10 μl of the test compound solution and incubated at room temperature for 10 min in the dark. Readings of the colored product (ferrous tripyridyltriazine complex) were then taken at 593 nm against ethanol. Results for the tested compounds are expressed as an increase in absorbance of the test sample compared to a sample containing the solvent. In the FRAP assay, the antioxidant potential of the sample was determined from a standard curve plotted using FeSO_4_·7H_2_O in the concentration range between 37.5 and 1200 μM

### Non-protein thiols (NPSH)

NPSH levels were determined using Ellman's method in which 5,5′-dithiobis(2-nitrobenzoic acid) (DTNB) was reduced by–SH group to 2-nitro-5-thiobenzoate (TNB) characterized by intensive yellow color, which showed maximum absorbance at 412 nm [13].

For determination of NPSH level, 950 μl of the plasma was first deprotonated by addition of 50 μl of cold 50% trichloroacetic acid, and then the sample was centrifuged at 10 000 x g at a temperature of +4°C for 10 min. To 850 μl of 0.2M phosphate buffer (pH 8.2), 100 μl of 6 mM DTNB and 50 μl of supernatant from deprotonated plasma were added. Absorbance was measured at a wavelength λ = 412 nm 1 min after supernatant addition. The total content of NPSH was determined from a standard curve prepared for the 1mM glutathione [14].

### DPPH radicle scavenging assay

The molecule of 1,1-diphenyl-2-picrylhydrazyl (α,α-diphenyl-β-picrylhydrazyl; DPPH) is characterized as a stable free radical by virtue of the delocalization of the spare electron over the molecule as a whole [15]. The delocalization also gives rise to the deep violet color (absorption in ethanol solution at about 520 nm). When DPPH is mixed with a substance that can donate a hydrogen atom, the solution loses the violet color (reduction). To the 20 μl of tested compounds 180 μl 0.2 mM DPPH reagent was added. The mixture was shaken vigorously and incubated at room temperature for 15 minutes, after that the absorbance was measured at 520 nm. Ascorbic acid and Trolox were used as references at concentrations identical to the experimental samples (100 μM, 50 μM and 10 μM).

### Cytotoxicity assay

#### Cell Culture

The human hepatocellular carcinoma cells Hep G2 [HEPG2] were obtained from the ATCC (HB8065). The cells were thawed according to the manufacturer's protocol form ATCC. HepG2 cells were cultivated in Eagle's Minimum Essential Medium (EMEM, ATCC) supplemented with 10% heat inactivated fetal bovine serum (ATCC), with added 100 IU/ml penicillin (ATCC) and 100 μg/ml streptomycin (ATCC). Cells were cultured in flasks with an area of 175 cm^2^, and incubated at 37°C, 5% CO_2_. For the test of compounds with the HepG2 cells line, hepatocytes were seed on 96-well culture plate at a density of 20 000 cells per well in fresh medium. Cells had grown for 24 hours in the incubator (37°C, 5% CO_2_) before performing experiments. After that dilutions of tested compounds were added and incubated for 24 hours in aseptic conditions.

#### Compounds

All compounds were dissolved in dimethyl sulfoxide (DMSO) with stock concentrations in the range of 10 mM. The compounds were incubated for 5 minutes with ultrasound in a water bath. From the stock, dilutions were prepared in phosphate buffered saline (PBS). All experiments were performed in triplicates, in two independent experiments.

#### Cell viability (PrestoBlue)

Cell viability was measured using the PrestoBlue reagent (Invitrogen). PrestoBlue reagent is a resazurin-based solution that functions as a cell viability indicator. Metabolically active cells are capable of reducing the PrestoBlue reagent, with the colorimetric changes used as an indicator to quantify the viability of cells in culture. This change can be determined by measuring the fluorescence. After 24 hours of incubation with the compounds PrestoBlue reagent was added to wells of a microplate in an amount equal to one tenth of the remaining medium volume. After 15 minutes of incubation at 37°C, the fluorescence intensity (Ex 530 Em 580 nm) was measured in a plate reader (POLARstar Omega, BMG Labtech). Viability values were calculated as a percentage of live cells with respect to the control sample (DMSO).

### The influence on the guinea pig ileum contraction induced by histamine

The experiment was performed according to the method described by Mogilski and colleagues [16]. A segment (15 cm) of male guinea pig ileum was excised from the small intestine and immersed into a Krebs solution (NaCl 120 mM, KCl 5.6 mM, MgCl_2_ 2.2 mM, CaCl_2_ 2.4 mM, NaHCO_3_ 19 mM, glucose 10 mM). The part of the ileum (5 cm) that was the closest to the ileocecal junction was removed. After 2 cm-long fragments of the ileum were cut, each of them was placed in 20 ml chamber of tissue organ bath system (Tissue Organ Bath System—750 TOBS, DMT, Denmark) filled with the Krebs solution at 37°C, pH 7.4, with constant oxygenation (O_2_/CO_2_, 19:1). The segments were stretched by means of closing clips between the metal rod and the force–displacement transducer. The preparations were allowed to stabilize in organ baths for 60 min under a resting tension of 0.5 g and were washed every 15 min with fresh Krebs solution. After the equilibration period a cumulative concentration–response curve was constructed for muscarinic receptor agonist: histamine (10 nM– 10 μM). Then the tissues were incubated with one of the concentrations of tested compounds for 15 min and the next cumulative concentration curve to the agonist was constructed. Only one concentration of the potential antagonist was tested in each piece of tissue. The experiment was repeated four to eight times.

### Data analysis

Results are presented as means ± S.E.M. In the experiments on the influence on blood pressure two-way ANOVA followed by Bonferroni test for multiple comparisons was used. In the biochemical studies comparisons between experimental and control groups were performed by one-way ANOVA, followed by Newman-Keuls *post hoc*. A value of p<0.05 was considered to be significant.

The concentrations of glucose, triglycerides, total cholesterol, HDL or LDL were calculated using the following equation:

$$cb=\frac{A_{b}\cdot cw}{A_{w}}$$

cb—glucose, triglycerides, total cholesterol, HDL or LDL concentration [mmol/l]

A_b_—sample’s absorbance

cw—standard’s concentration

A_w_—standard’s absorbance

Liver enzymes activities were determined using the following equations:

$$ASAT=\frac{V_{t}\cdot 10^{5}}{\varepsilon \cdot l\cdot V_{s}}\cdot \Delta A/min$$

ASAT—aspartate aminotransferase activity [U/l]

V_t_—total volume of reaction mixture

*ε—*molar extinction coefficient of NADH at the wavelength 340 nm– 6.3x10^2^ m^2^/mol

l—light path length

V_s_−sample’s volume

$$ALAT=\frac{V_{t}\cdot 10^{5}}{\varepsilon \cdot l\cdot V_{s}}\cdot \Delta A/min$$

ALAT—alanine aminotransferase activity [U/l]

V_t_—total volume of reaction mixture

*ε—*molar extinction coefficient of NADH at the wavelength 340 nm– 6.3x10^2^ m^2^/mol

l—light path length

V_s_—sample’s volume

$$GGT=\frac{V_{t}\cdot 10^{5}}{\varepsilon \cdot l\cdot V_{s}}\cdot \Delta A/min$$

GGT– γ-glutamyl transferase activity [U/l]

V_t_−total volume of reaction mixture

*ε –*molar extinction coefficient of 5-amino-2-nitrobenzoate at the wavelength 405 nm– 950 m^2^/mol

l—light path length

V_s_−sample’s volume

The radical scavenging activity was calculated as follows:

$$scavenging\;rate=\frac{A_{s}-A_{i}}{A_{s}}\cdot 100$$

A_s_−DPPH absorbance

A_i_−the absorbance of DPPH in the presence of various extracts.

The results obtained in the cytotoxicity assay were normalized to the control sample (DMSO), before blank corrected (medium), wherein the intensity of fluorescence was taken as 100%.

In functional experiments pK_B_ value was estimated using the following equation [17]:

$$pK_{B}=-log_{10}\frac{B}{DR-1}$$

Where B is the molar antagonist concentration and DR is the ratio between the EC_50_ of the agonist in the presence and absence of the antagonist. pK_B_ value is equivalent to the pA_2_ value and was calculated if only one concentration of tested compound was effective.

## Results

### HBK-14 and HBK-15 did not influence blood pressure after chronic treatment

Neither chronic treatment with HBK-14 nor HBK-15 influenced blood pressure in normotensive rats (Table 1). Terazosin (5 mg/kg) significantly reduced systolic and diastolic blood pressure since the 3^rd^ measurement (Table 1).

**Table 1:** The influence of studied compounds on systolic and diastolic blood pressure after chronic treatment in rats.

Treatment	Dose (mg/kg)	Blood pressure	Measurement					
			0	1	2	3	4	5
**Vehicle**	-	systolic	136.8 ± 5.2	141.8 ± 4.0	133.7 ± 4.6	144.5 ± 6.9	144.0 ± 5.5	145.5 ± 3.8
		diastolic	103.8 ± 6.0	115.3 ± 5.0	104.3 ± 4.7	106.8 ± 7.5	107.0 ± 3.7	115.0 ± 3.9
**HBK-14**	2.5	systolic	138.2 ± 6.0	144.7 ± 4.8	134.8 ± 5.6	142.0 ± 3.7	138.3 ± 5.5	142.8 ± 7.2
		diastolic	105.2 ± 9.2	115.2 ± 8.7	108.5 ± 5.8	110.3 ± 4.7	111.3 ± 2.9	113.3 ± 5.8
	5	systolic	140.0 ± 3.0	131.5 ± 2.0	132.3 ± 4.5	141.3 ± 5.6	134.3 ± 4.8	143.5 ± 5.7
		diastolic	111.3 ± 4.6	107.5 ± 3.0	112.0 ± 6.8	111.5 ± 3.9	106.2 ± 3.1	116.3 ± 3.9
**Vehicle**	-	systolic	139.0 ± 3.0	140.4 ± 2.6	136.2 ± 1.8	136.8 ± 4.2	136.6 ± 3.5	138.0 ± 2.8
		diastolic	105.2 ± 3.3	108.2 ± 2.2	104.2 ± 3.4	108.4 ± 4.9	106.4 ± 4.2	108.6 ± 2.8
**HBK-15**	1.25	systolic	133.4 ± 3.5	133.0 ± 4.9	133.2 ± 5.6	135.6 ± 4.9	139.4 ± 3.3	135.6 ± 5.8
		diastolic	103.4 ± 3.7	102.8 ± 2.0	106.6 ± 3.4	104.6 ± 5.2	110.4 ± 4.9	103.6 ± 1.7
	5	systolic	134.0 ± 4.2	132.2 ± 3.0	132.2 ± 4.9	133.8 ± 2.3	136.0 ± 2.6	139.2 ± 2.5
		diastolic	105.0 ± 4.6	101.8 ± 4.3	105.0 ± 4.4	105.6 ± 5.2	97.6 ± 4.5	102.2 ± 5.4
**Vehicle**	-	systolic	134.3 ± 1.8	139.8 ± 2.7	132.5 ± 3.2	137.3 ± 4.1	134.8 ± 6.0	137.0 ± 4.4
		diastolic	107.8 ± 4.1	114.0 ± 2.3	107.2 ± 4.4	107.0 ± 7.1	110.7 ± 4.4	104.5 ± 3.0
**Terazosin**	5	systolic	128.4 ± 2.9	122.6 ± 1.7	117.0 ± 3.8	115.2 ± 2.9*	114.0 ± 1.5*	111.6 ± 2.7**
		diastolic	108.0 ± 3.7	102.4 ± 3.7	100.2 ± 3.6	90.0 ± 2.8*	88.6 ± 3.4**	85.2 ± 4.6**

HBK-14 (2.5 and 5 mg/kg), HBK-15 (1.25 and 5 mg/kg), terazosin (5 mg/kg) or saline (vehicle-treated groups) were administered intraperitoneally to normotensive rats for 14 days. During that time the blood pressure was measured using a non-invasive blood pressure measurement system for rodents. The blood pressure was measured before the first injection of the compound on the 1^st^ day (0) and on the 3^rd^ (1), 5^th^ (2), 8^th^ (3), 11^th^ (4) and 14^th^ (5) day of compound administration. The data are the means ± S.E.M. Statistical analysis: two-way ANOVA test with repeated measurements, Bonferroni *post hoc* test.

*p<0.05,

**p<0.01 *vs* measurement 0; n = 5–6 rats per group.

### HBK-14 and HBK-15 did not influence lipid profile after chronic treatment

None of the compounds influenced total cholesterol, LDL, HDL and triglycerides level after 14 days of injection (Table 2).

**Table 2:** The influence of HBK-14 and HBK-15 on lipid and carbohydrate profile after chronic administration in rats.

Treatment	Dose (mg/kg)	Total cholesterol [mmol/l]	LDL [mmol/l]	HDL [mmol/l]	Triglycerides [mmol/l]	Glucose [mmol/l]
**Vehicle**	-	1.72 ± 0.19	1.10 ± 0.09	0.62 ± 0.08	0.17 ± 0.03	5.50 ± 0.27
**HBK-14**	2.5	1.70 ± 0.09	1.01 ± 0.09	0.59 ± 0.05	0.16 ± 0.03	5.53 ± 0.23
	5	1.75 ± 0.10	1.11 ± 0.10	0.63 ± 0.07	0.13 ± 0.02	5.50 ± 0.23
**Vehicle**	-	1.62 ± 0.05	0.96 ± 0.08	0.60 ± 0.05	0.16 ± 0.01	5.27 ± 0.59
**HBK-15**	1.25	1.68 ± 0.16	1.00 ± 0.08	0.57 ± 0.03	0.16 ± 0.04	5.36 ± 0.22
	2.5	1.52 ± 0.09	0.94 ± 0.03	0.61 ± 0.05	0.18 ± 0.01	4.56 ± 0.23

HBK-14 (2.5 and 5 mg/kg), HBK-15 (1.25 and 5 mg/kg) or saline (vehicle-treated groups) were administered intraperitoneally to normotensive rats for 14 days. Following the last administration, rodents were deprived of food. 24 hours later rats were anaesthetized, heparinized and their plasma was collected. The data are the means ± S.E.M. Statistical analysis: one-way ANOVA test with repeated measurements, Newman-Keuls *post hoc* test. n = 4–6 rats per group.

### HBK-14 and HBK-15 did not influence glucose level after chronic treatment

The studied compounds did not influence glucose level in rat plasma after chronic treatment (Table 2).

### HBK-14 and HBK-15 did not influence liver enzymes activity after chronic treatment

Neither HBK-14 nor HBK-15 influenced the activity of ALAT, ASAT and GGT after chronic treatment in rats (Table 3).

**Table 3:** The influence of HBK-14 and HBK-15 on liver enzymes activity.

Treatment	Dose (mg/kg)	ALAT [U/l]	ASAT [U/l]	GGT [U/l]
**Vehicle**	-	57.04 ± 2.90	74.63 ± 9.02	9.234 ± 1.94
**HBK-14**	2.5	49.78 ± 2.26	76.08 ± 12.76	11.28 ± 1.66
	5	48.58 ± 5.28	78.53 ± 7.73	9.44 ± 0.62
**Vehicle**	-	59.75 ± 5.25	71.00 ± 5.99	11.50 ± 1.42
**HBK-15**	1.25	62.70 ± 4.85	71.06 ± 6.19	12.97 ± 1.81
	2.5	69.19 ± 7.23	75.95 ± 4.39	11.32 ± 1.14

HBK-14 (2.5 and 5 mg/kg), HBK-15 (1.25 and 5 mg/kg) or saline (vehicle-treated groups) were administered intraperitoneally to normotensive rats for 14 days. Following the last administration, rodents were deprived of food. 24 hours later rats were anaesthetized, heparinized and their plasma was collected. The data are the means ± S.E.M. Statistical analysis: one-way ANOVA test with repeated measurements, Newman-Keuls *post hoc* test. n = 4–6 rats per group.

### HBK-14 and HBK-15 did not influence ferric reducing ability of plasma after chronic treatment

Chronic administration of studied compounds did not influence the ferric reducing ability of plasma (Table 4).

**Table 4:** The influence of chronic treatment with HBK-14 or HBK-15 on ferric reducing ability of plasma (FRAP) and non-protein thiols (NPSH) in rats.

Treatment	Dose (mg/kg)	FRAP [μmol/l]	NPSH [mmol/l]
**Vehicle**	-	1.60 ± 0.16	0.66 ± 0.05
**HBK-14**	2.5	1.38 ± 0.07	0.65 ± 0.01
	5	1.29 ± 0.07	0.62 ± 0.03
**Vehicle**	-	1.83 ± 0.05	0.63 ± 0.02
**HBK-15**	1.25	1.93 ± 0.04	0.62 ± 0.02
	2.5	1.83 ± 0.06	0.65 ± 0.01

HBK-14 (2.5 and 5 mg/kg), HBK-15 (1.25 and 5 mg/kg) or saline (vehicle-treated groups) were administered intraperitoneally to normotensive rats for 14 days. Following the last administration, rodents were deprived of food. 24 hours later rats were anaesthetized, heparinized and their plasma was collected. The data are the means ± S.E.M. Statistical analysis: one-way ANOVA test with repeated measurements, Newman-Keuls *post hoc* test. n = 4–6 rats per group.

### HBK-14 and HBK-15 did not induce any changes in non-protein thiols after chronic treatment

The repeated administration of tested compounds did not induce any changes in protein -SH group levels in plasma (Table 4).

### HBK-14 and HBK-15 did not inhibit DPPH

None of the compounds at 100 μM showed antioxidant properties, whereas vitamin C and Trolox (reference compounds) at the same concentration counteracted the oxidation by 90% and 91%, respectively (Table 5).

**Table 5:** DPPH scavenging effects of HBK-14 and HBK-15.

Treatment	% Inhibition of DPPH at various concentrations of studied compounds		
	100 μM	50 μM	10 μM
**HBK-14**	3.0 ± 0.7	3.0 ± 0.3	2.0 ± 0.3
**HBK-15**	4.0 ± 0.9	4.0 ± 0.3	5.0 ± 0.3
**Ascorbic acid**	90.0 ± 0.3	73.0 ± 2.0	14.0 ± 1.7
**Trolox**	91.0 ± 0.3	84.0 ± 2.0	17.0 ± 1.5

HBK-14 and HBK-15 were tested at three concentrations (100 μM, 50 μM, 10 μM). The level of DPPH radical was measured spectrophotometrically at 520 nm. Values are represented as mean± S.E.M (n = 3) (percentage of DPPH inhibition).

### HBK-14 and HBK-15 did not induce cytotoxicity

Both compounds were cytotoxic at the concentration of 50 μM, whereas at the concentration 10 μM only HBK-15 did not show cytotoxic properties (Table 6). None of compounds possessed cytotoxic properties at the concentration 1 μM.

**Table 6:** Cytotoxicity studies for HBK-14 and HBK-15.

Compound	Concentration (μM)	Viability of cells (%)
**HBK-14**	50	59.0 ± 9.5
	10	63.0 ± 0.0
	1	79.0 ± 0.0
**HBK-15**	50	71.0 ± 2.0
	10	99.0 ± 6.5
	1	101.0 ± 1.0

Cell viability in the Hep G2 cell line after exposure to HBK-14 and HBK-15. Compounds were tested at three concentrations (50 μM, 10 μM, 1 μM) for 24 hours. Values are represented as mean± S.E.M (n = 3) (percentage of control).

### HBK-14 and HBK-15 possessed very week antihistaminic properties

**Fig 2:**
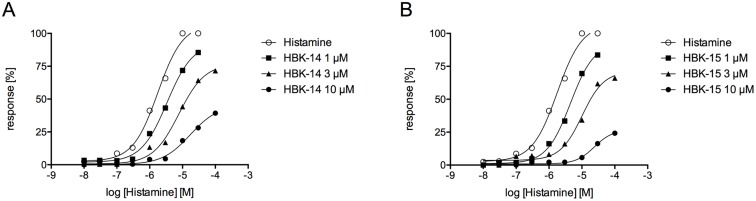
The effect of HBK-14 and HBK-15 on histamine H_1_ receptor in isolated guinea pig ileum. Concentration–response curves to histamine in the absence or presence of increasing concentrations of HBK-14 or HBK-15. The results are expressed as the percentage of maximal response to histamine in the corresponding concentration–response curve. Each point represents the mean ± S.E.M (n = 4–8).

Histamine induced concentration-dependent guinea pig ileum contractions; the pEC_50_ value (negative logarithm of the agonist concentration at which the response reached 50% of the maximal response) was 5.75 ± 0.05 (Fig 2). Neither HBK-14 nor HBK-15 alone contracted the ileum (data not shown). HBK-14 (1 μM) and HBK-15 (1 μM) slightly shifted histamine-induced contractions (Fig 2), which suggested a competitive antagonism. However, at the concentration 3 and 10 μM both compounds decreased the maximum effect of histamine. HBK-14 decreased the response by 28% and 58%, and HBK-15 by 34% and 75%, respectively. This suggested a non-competitive antagonism. The affinity expressed as the pK_B_ was 5.96 ± 0.01 for HBK-14 and 6.19 ± 0.01 for HBK-15.

## Discussion

We found that chronic administration of dual 5-HT_1A_ and 5-HT_7_ antagonists with α_1_-adrenolytic properties i.e. HBK-14 and HBK-15 did not influence blood pressure, lipid profile, glucose level or liver enzymes activity in rats. We also demonstrated that neither HBK-14 nor HBK-15 possessed antioxidant or cytotoxic properties at antidepressant- and anxiolytic-like doses. HBK-14 and HBK-15 showed very weak and thus negligible antihistaminic properties.

Older generation of antidepressants (i.e. tricyclic antidepressants) show cardiovascular toxicity. To overcome this, pharmaceutical companies introduced selective serotonin reuptake inhibitors. Surprisingly, many studies suggested that their use was associated with prolonged QTc interval, arrhythmias, and orthostatic hypotension (for review see [18]). Fuller and colleagues [19] demonstrated that fluoxetine had antihypertensive properties in rats. Similarly, Cherin and colleagues [2] showed that the drug increased the risk of syncope and orthostatic hypotension in elder patients. Altogether, the data suggest that antidepressants are still not devoid of the negative effect on cardiovascular system, and therefore this aspect should be carefully examined in the case of drug candidates.

We previously demonstrated that dual 5-HT_1A_ and 5-HT_7_ antagonists (HBK-14 and HBK-15), which showed significant antidepressant- and anxiolytic-like activities [7], possessed α_1_-adrenolytic properties and lowered blood pressure in rats [8]. HBK-14 (but not HBK-15) showed hypotensive activity at antidepressant-like doses after acute treatment [8]. None of the compounds influenced heart rate or QTc interval after acute administration in rats [8]. Since antidepressants are administered chronically, we evaluated the effects of both compounds on blood pressure after chronic treatment in rats. As the studied compounds possessed α_1_-adrenolytic properties, we compared them with terazosin (α_1_-adrenolytic drug). Our studies revealed that neither HBK-14 nor HBK-15 influenced blood pressure, whereas terazosin showed significant hypotensive effect in normotensive rats since the 3^rd^ measurement (after one week of administration).

Interestingly, HBK-14 possessed strong hypotensive properties at antidepressant- and anxiolytic-like doses after acute treatment [8], but we did not observe such effect after chronic administration. This might be due to the complexity of blood pressure regulation. Three main mechanisms regulate blood pressure: global, local and renal-endocrine system. Globally, autonomic nervous system (sympathetic and parasympathetic) controls blood flow. Local mechanisms include acute and chronic vasoconstriction, change in the number and caliber of blood vessels supplying the tissue and endothelial autocrine secretions, whereas renal-endocrine system regulates body fluids and salt homeostasis. Thus, we cannot always interpolate the results obtained in experimental models such as isolated arteries (tail artery, aorta) to the whole systemic vasculature. Therefore, we think that in case of HBK-14, the reduced blood pressure might have been compensated by one of the above mechanisms. Other explanations include only temporary reduction in blood pressure or short t_0.5_ of HBK-14, but this issue requires pharmacokinetic studies. Nevertheless, we believe that the studied compounds have beneficial pharmacological profiles i.e. significant antidepressant- and anxiolytic-like properties and no influence on blood pressure after chronic treatment (despite α_1_-adrenolytic activity).

The data concerning the effect of antidepressants on lipid homeostasis are ambiguous (reviewed in [20]). While chronic treatment with fluoxetine, sertraline, fluvoxamine and citalopram had no effect [21], amitriptyline and nortriptyline significantly increased triglycerides levels in depressed patients [22,23]. Venlafaxine and paroxetine administered chronically significantly increased the levels of LDL and total cholesterol in patients suffering from panic disorders, and healthy males, respectively [1,24]. In depressed individuals chronic treatment with imipramine significantly lowered HDL level [25]. Conversely, in obese patients bupropion administered for 48 weeks caused an increase in HDL level [26]. Altogether, these findings suggest that some antidepressants may have a negative impact on lipid profile.

Bearing that in mind, we evaluated the influence of 2-week administration of studied compounds on the levels of triglycerides, total cholesterol, HDL and LDL in rat’s plasma. We showed that neither HBK-14 nor HBK-15 influenced lipid profile. On one hand, some data suggest that in depressed patients the level of LDL is lower [27], and its increase might be beneficial [28]. However, Liang and colleagues showed opposite results. The Authors proved that LDL levels in depressed individuals were not lower, but even higher that in healthy individuals [29]. Since the data concerning the lipid profile in depressed individuals are inconsistent, we believe that the lack of effect of HBK-14 and HBK-15 on triglycerides, total cholesterol, LDL or HDL levels is beneficial.

Depressed patients often show impaired glucose tolerance and an increased risk to develop diabetes mellitus [30,31]. Moreover, antidepressants affect glucose homeostasis (for review see [32]). Erenmemisoglu and colleagues [33] found that in mice tricyclic antidepressants might cause hyperglycemia, while selective serotonin reuptake inhibitors might reduce plasma glucose independently of insulin levels. Similarly, Gupta and colleagues [34] reported that acute, but not chronic, treatment with imipramine increased blood glucose levels in rabbits. Altogether, these findings suggest that noradrenergic antidepressants might cause hyperglycemia, while some serotonergic agents might enhance insulin sensitivity and reduce hyperglycemia.

Since HBK-14 and HBK-15 affect both serotonergic and adrenergic receptors, we investigated their effect on glucose levels after chronic treatment. Our results show that neither HBK-14 nor HBK-15 influenced glucose levels in euglycemic rats, which in our opinion is beneficial. Both an increase and a decrease in blood glucose levels in non-diabetic patients are not desirable.

A central organ of xenobiotics biotransformation—liver—is particularly prone to drug toxicity. Voican and colleagues [35] indicated amitriptyline, bupropion, duloxetine, imipramine, iproniazid, phenelzine, nefazodone, trazodone, and tianeptin as drugs with increased risk of liver injury. In contrast, Gahr and colleagues [36] in their review did not report bupropion as hepatotoxic. Both research teams, however, indicated citalopram, escitalopram, paroxetine and fluvoxamine as the least hepatotoxic antidepressants.

Our experiments revealed that chronic treatment with neither HBK-14 nor HBK-15 caused liver injury. Both ALAT and ASAT activities were comparable to control groups, which clearly indicates that at antidepressant- and anxiolytic-like doses the studied compounds did not cause hepatotoxicity. Moreover, the activity of GTT, which could be found among others in the liver, was also comparable to control groups. This suggests that 2-week treatment with studied compounds did not injure the liver.

Drug-induced liver injury excludes drug candidates from later stages of drug development. Thus, early detection of drug-induced hepatotoxicity saves time and resources. Human hepatocarcinoma cell line Hep G2 is one of the models used across the pharmaceutical and chemical industries to detect hepatotoxicity induced by new compounds [37]. In our experiments on Hep G2, HBK-15 at the concentration of 1 μM did not cause significant cell death, whereas HBK-14 inconsiderably reduced cell viability. This suggests that neither HBK-14 nor HBK-15 were hepatotoxic at this concentration, which is in agreement with the above results. Moreover, it proves that intrinsic activity of studied compounds, evaluated in our previous experiments [7], was not due to their cytotoxic effect.

Since the ability to scavenge free radicals, would be a desirable feature of a drug candidate, we determined antioxidant properties of HBK-14 and HBK-15 after 2-week treatment. Our experiments showed that none of the studied compounds showed antioxidant properties in FRAP or NPSH assays. DPPH radical scavenging activity studies confirmed our findings. Neither HBK-14 nor HBK-15 at the concentration of 100 μM showed antioxidant properties in this assay. The absorbance values were comparable to vehicle (DMSO). This is in agreement with our previous studies, where we demonstrated that HBK-14 and HBK-15 did not show antioxidant activity [8].

The blockade of histaminic H_1_ receptors in the central nervous system might cause various unwanted effects, including dizziness, anxiety or increased appetite leading to weight gain. Therefore, we investigated potential antihistaminic properties of the studied compounds using isolated guinea pig ileum model. Our experiments revealed that HBK-14 and HBK-15 were very weak H_1_ receptor antagonists. Previous experiments performed in our laboratory showed that pA_2_ value for antazoline (antihistaminic drug) was 7.141 [16]. This value was much higher than pK_B_ values obtained for HBK-14 (5.75) or HBK-15 (5.96), thus we concluded that the studied compounds possessed negligible antihistaminic properties.

The limitation of our study was a relatively short period of drug administration i.e. 2 weeks. Therefore, in future experiments we plan to extend the treatment to 4–6 weeks using depression model (e.g. chronic mild stress). Since many patients with diabetes and metabolic syndrome suffer from depression or metabolic syndrome, we also think that the effect of HBK-14 and HBK-15 should be investigated in diabetic rats (e.g. diabetes induced chemically by streptozocin) and animals with increased triglycerides and LDL levels (e.g. diet-induced or Zucker rats), as well as spontaneously hypertensive rats.

## Conclusions

We demonstrated that 2-week treatment with dual 5-HT_1A_ and 5-HT_7_ antagonists with α_1_-adrenolytic properties i.e. HBK-14 and HBK-15 did not influence blood pressure, lipid and carbohydrate profiles or liver enzymes activity in normotensive rats. We also showed that neither HBK-14 nor HBK-15 possessed antioxidant or cytotoxic properties. Positive results of our preliminary experiments on the safety of HBK-14 and HBK-15 encourage further studies on their effectiveness in the treatment of depression and/or anxiety.
